# Cognitive outcome of classic infantile Pompe patients receiving enzyme therapy

**DOI:** 10.1186/1471-2474-14-S2-P14

**Published:** 2013-05-29

**Authors:** BJ Ebbink, FK Aarsen, CM van Gelder, JMP van den Hout, N Weisglas-Kuperus, J Jaeken, MH Lequin, WFM Arts, AT van der Ploeg

**Affiliations:** 1 Center for Lysosomal and Metabolic Diseases, Erasmus MC University Medical Center, Rotterdam, The Netherlands

## Objective

Classic infantile Pompe disease affects many tissues, including the brain. Untreated infants die within their first year. While enzyme-replacement therapy (ERT) significantly increases survival, its potential limitation is that the drug cannot cross the bloodbrain- barrier. We therefore investigated long-term cognitive development in patients treated with ERT.

## Methods

We prospectively assessed cognitive functioning in 10 children with classic infantile Pompe disease who had been treated with ERT since 1999. Until 2004, infants and young children were assessed with the Bayley Scales of Infant Development (BSID-II; number of tests = 23). After 2004, we switched to the Griffiths Mental Developmental Scales (Griffiths; number of tests = 19), expecting it to differentiate better between various domains. Older children were assessed using the Wechsler Intelligence Scales for Children (WISC-III; number of tests = 5). For children with tetraplegia, we used the Raven Colored or Standard Progressive Matrices (number of tests = 3). For those with impaired hearing, we used the Snijders Oomen Nonverbal Intelligence test-Revised (SON-R 2½-7, number of tests = 1). In total, 51 tests were performed. Brain imaging was performed in six children.

## Results

During the first four years of life, developmental scores in 10 children ranged from above average development to severe developmental delay; they were influenced by the type of intelligence test used, severity of motor problems, speech/language difficulties and age at start of therapy. Five of the children were also tested from five years onwards. Among them were two tetraplegic children whose earlier scores had indicated severe developmental delay. These scores now ranged between normal and mild developmental delay, and indicated that at young age poor motor functioning may interfere with proper assessment of cognition. We found delayed processing speed in two children. Brain imaging revealed periventricular white-matter abnormalities in four.

## Conclusion

Cognitive development at school age ranged between normal and mildly delayed in our long-term survivors with classic infantile Pompe disease treated with ERT. The oldest was 12 years. We found that cognition is easily underestimated in children under five with poor motor functioning.

